# Online Inductance Monitoring Based on Dynamic Characteristics and ESR Effect Compensation for Buck Converter Without Current Sensor

**DOI:** 10.3390/s25123589

**Published:** 2025-06-06

**Authors:** Chen Chen, Liang Wang, Wanyang Wang, Run Min, Qiaoling Tong

**Affiliations:** 1 School of Electronic Information, Wuhan University of Science and Technology, Wuhan 430081, China; 2 China Nuclear Power Operation Technology Corporation, Ltd., Wuhan 430073, China; 3 School of Integrated Circuit, Huazhong University of Science and Technology, Wuhan 430074, China

**Keywords:** DC-DC converter, inductance, condition monitoring, current mode control

## Abstract

Inductor parameter variations often affect the control performance of digital current mode (CM)-controlled buck converters as their high performance relies on accurate converter modeling. However, recent studies have shown that reliably monitoring inductance with current sensors and high-frequency sampling greatly increases the overall cost of this process. To address this issue, an online inductance monitoring method without a current sensor is proposed in this study. First, an inductance calculation model is derived by applying the dynamic characteristics of a buck converter with inductor volt-second and capacitor charge balance principles. The model’s accuracy is guaranteed by considering inductor current switching ripple characteristics. Nevertheless, output capacitor equivalent series resistance (ESR) can degrade the accuracy of the proposed calculation model. Thus, to enhance the tolerance of the inductance calculation model to capacitor ESR, the ESR effect on inductance monitoring is investigated. With the proposed capacitor ESR estimation method, inductance monitoring achieves reliable accuracy, even for a buck converter with high capacitor ESR. The effectiveness of the proposed method is verified by simulations and experiments on a buck converter with digital sensorless current mode (SCM) control.

## 1. Introduction

Switch-mode DC-DC converters have been widely used in portable electronic devices, renewable energy systems, and aerospace fields due to their small size, light weight, and high efficiency [1,2,3,4,5]. To improve their dynamic performance, several digital current mode (CM) control strategies have been investigated, such as digital peak/average/valley predictive current control (PCC) [6,7,8], model predictive control [9,10], and hybrid ripple-based control [11]. Although stability and transient response capabilities are continuously improving, the control performance of these algorithms still relies on accurate inductor current sensing. There are two commonly used current sensing methods: the sampling resistor and hall-effect sensor methods [12,13]. Utilizing sampling resistors is a simple approach, but the accuracy and the induced power loss should be considered. Hall-effect sensors can provide high accuracy and low loss. However, increased costs and delays constrain their application.

To solve these issues, inductor current estimation algorithms have been investigated for DC-DC converters [14,15,16,17,18]. Based on inductor volt-second characteristics, a simple current estimator for digital sensorless current mode (SCM) control is proposed in [14]. However, since component parasitic parameters are ignored, the estimation error increases linearly with time, leading to output voltage steady state error. In addition to component parasitics, other factors can degrade current estimation accuracy, such as output voltage sampling errors, switching delay, and inductance variation. Considering inductor ESR and the conduction resistance of switching components, the estimation error converges to a constant value, and the output voltage steady state error is eliminated [15]. The compensation strategy for current estimators with sensorless PCC is improved by investigating parasitic parameters and output voltage sampling errors in detail [16,17]. In [18], the effect of switching delay on inductance estimation was eliminated by tracking the width of the switch node voltage. That study improved current estimation accuracy and control performance, where accurate inductance is always required. Since inductance can be affected by many factors, such as switching frequency, temperature, current, and aging, it should be precisely acquired.

To track inductance variation accurately with low complexity and cost, a series of online monitoring methods are investigated in [19,20,21,22,23,24]. In [19], a look-up-table-based method was constructed to compensate for the effect of DC-bias current on inductance. It was easy to implement, but the monitoring accuracy was difficult to guarantee since the influence of the aforementioned factors was ignored. In [20], a least mean squares algorithm based on a continuous time model was proposed. However, twenty-five samples for voltages and inductor current are required during one switching cycle, increasing the overall complexity. Aiming to address this issue, an adaptive identifier for inductance monitoring was proposed with a generalized gradient descent algorithm [21]. To further improve the convergence rate of parameter estimation, an adaptive model observer-based inductance and capacitance monitoring scheme was investigated [22]. However, the sampling frequency of the current and voltage is still much higher than the switching frequency. Other inductance and capacitance monitoring techniques were proposed in [23,24], embedding the particle swarm optimization algorithm into the mathematical model. Although the accuracy of inductance estimation is gradually improving, common issues need to be addressed in practice:

(1) The demand for accurate and high-speed current sensors increases the hardware cost and complexity.

(2) High-frequency voltage/current sampling is needed, which is much higher than switching frequency. This greatly increases costs and is hard to implement in high switching frequency applications.

(3) Iterative calculations increase the requirements for digital signal processors.

To solve these issues, this study proposes a current-sensorless inductance monitoring method based on dynamic characteristics. This monitoring technique utilizes dynamic line/output voltages, where a small and short pulse excitation signal is imposed on the reference voltage. By combining the volt-second balance principle and inductor current switching ripple characteristic of the dynamic process, an inductance calculation model is derived, where the dynamic inductor current characteristics are required. In the calculation model, the difference in the inductor average currents in two neighboring switching cycles is estimated by using the capacitor charge balance principle. Therefore, only one sampling for line and output voltages is required per switching cycle. Furthermore, the proposed calculation model is investigated with respect to output capacitor equivalent series resistance (ESR). The results indicate that the inductor monitoring error is proportional to the ESR, which should be fully considered in aging and certain high ESR situations. To compensate for the ESR effect, an ESR estimation method is derived by revising the original inductance derivation process. According to this approach, reliable inductance monitoring accuracy is ensured even for systems with high capacitor ESR.

This study is organized as follows. In Section 2, the structure of an SCM control law with online inductance monitoring is introduced. The precondition for inductance calculation is created by periodically changing the reference voltage with a small amplitude. Then, an inductance calculation model based on dynamic characteristics is proposed, which is current-sensorless. In Section 3, to eliminate the monitoring error caused by output capacitor ESR, the inductance calculation model is re-derived considering capacitor ESR. Furthermore, an ESR estimation method is proposed to guarantee the accuracy of inductance monitoring. The necessity of inductance monitoring is proved by simulations in Section 4. The experimental results and corresponding analysis are presented in Section 5. Finally, a conclusion is provided in Section 6.

## 2. Online Inductance Monitoring Method for Buck Converter

### 2.1. SCM Control with Online Inductance Monitoring

To obtain well-regulated dynamic responses, an online inductance monitoring method is proposed to update the preset inductance in an SCM controller. Since inductance is the key parameter of SCM controllers, it is necessary to track inductance variation using the online monitoring method.

**Figure 1:**
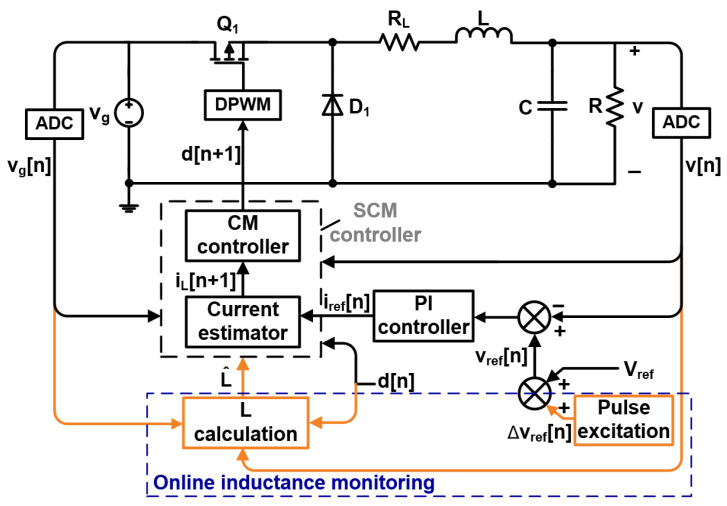
Block diagram of SCM-controlled buck converter with proposed online inductance monitoring method.

Figure 1 illustrates a block diagram of the proposed online inductance monitoring method. It consists of a MOSFET switch, *Q*_1_; a diode, *D*_1_; an inductor, *L*; inductor equivalent series resistance, *R_L_*; an output capacitor, *C*; and a load resistor, *R*. The line and output voltages used in the digital controller and online inductance monitoring module are sampled by AD converters (ADCs).

Without current sensors or additional AD converters, the inductance is calculated according to the sampled line output, *v_g_*[*n*]; the output voltage, *v*[n]; and the duty cycle, *d*[n]. The sampling frequency of line and output voltages follows the switching frequency, *T*. The reference current, *i_ref_*, is provided by the outer voltage loop. In the inner compensation loop, inductor current, *i_L_*, is calculated by a current estimator. The duty cycle, *d*, is generated by the current mode (CM) controller, which eliminates the error between *i_ref_* and *i_L_*. With a digital pulse width modulator (DPWM), the duty cycle is converted into the driving signal of the MOSFET switch, *Q*_1_.

The current estimator and CM controller are provided in (1) and (2). Their detailed descriptions are shown in Appendix A.1.

(1) $$i_{L}(n+1)=i_{L}(n)+\frac{T}{L}[v_{g}(n)d(n)-v(n)-i_{L}(n)R_{L}],$$

(2) $$d(n+1)=\frac{L[i_{ref}(n)-i_{L}(n+1)]+[v(n)+i_{L}(n)R_{L}]T}{v_{g}(n)T}.$$

For the proposed online inductance monitoring method, a pulse excitation signal with small amplitude and short duration is used to periodically change the reference voltage, *v_ref_*, which provides a required dynamic state. The influence of temperature, aging, and other factors on the parameters is ignored since the inductance is regarded as a constant value in the following calculation process. Based on the dynamic characteristics with inductor volt-second balance and capacitor charge balance principles, the inductance calculation model can be derived.

### 2.2. Inductance Calculation Model Based on Dynamic Characteristics

**Figure 2:**
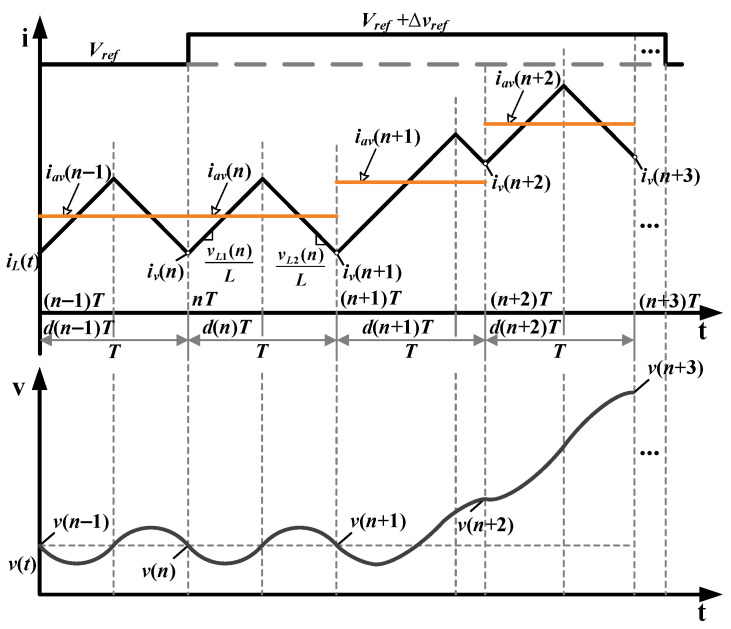
Waveforms of inductor current and output voltage when small amplitude pulse is injected.

The inductor current and output voltage during the pulse excitation process are illustrated in Figure 2. The reference voltage changes from *V_ref_* to *V_ref_* + Δ*v_ref_* at the beginning of the *n*th switching cycle. With the employed CM control, the duty cycle of the *n*th switching cycle is calculated in the previous cycle. The duty cycle changes from the (*n* + 1)th cycle, which introduces a dynamic state.

Inductance monitoring is built based on a converter model. For the traditional model indicated by (1), the inductor current in the switching cycle is considered a constant value, ignoring the current switching ripple effects. However, since the current ripple ratio is usually designed to be around 0.4 in most CCM operations, the induced modeling error will reduce the accuracy of the inductance online calculation.

To eliminate the modeling error, an improved discrete-time inductor current equation for the buck converter under trailing edge modulation is derived by

(3) $$i_{v}(n+1)-i_{v}(n)=\frac{T}{L}\left[v_{L1}(n)d(n)-v_{L2}(n)d^{\prime }(n)\right],$$

where $i_{v}(n)$ represents the valley inductor current of the *n*th switching cycle. $v_{L1}(n)$ and $v_{L2}(n)$ denote the inductor voltage during switching on and off, respectively, shown as follows:

(4) $$v_{L1}(n)=v_{g}(n)-v(n)-i_{av}(n)R_{L},$$

(5) $$v_{L2}(n)=v(n)+i_{av}(n)R_{L}.$$

Substituting (4) and (5) into (3) provides

(6) $$L\frac{i_{v}(n+1)-i_{v}(n)}{T}=v_{g}(n)d(n)-v(n)-i_{av}(n)R_{L}=v_{L}(n),$$

where $v_{L}(n)$ and $i_{av}(n)$ denote the inductor voltage and average inductor current of the *n*th switching cycle, respectively. Compared with (1), the inductor current in the switching cycle is referred to as *i_v_* and *i*_a*v*_, explaining the detailed characteristics of the inductor current ripple. Accordingly, the relationship between $i_{v}(n)$ and $i_{av}(n)$ is provided by

(7) $$\begin{array}{cc} i_{v}(n) & =i_{av}(n)-\frac{T}{2L}\left[v_{L1}(n)d(n)d^{\prime }(n)+v_{L1}(n)d(n)-v_{L2}(n){d^{\prime }}^{2}(n)\right]\\ & =i_{av}(n)-\frac{v_{x}(n)}{L}, \end{array}$$

where $v_{x}(n)=[v_{L1}(n)d(n)d^{\prime }(n)+v_{L1}(n)d(n)-v_{L2}(n){d^{\prime }}^{2}(n)]T/2$. The derivation is provided in Appendix A.2.

Substituting (7) into (6), the inductance calculation model based on the dynamic characteristic of the inductor volt-second balance principle is derived by

(8) $$\hat{L}=\frac{v_{x}(n+1)-v_{x}(n)+v_{L}(n)T}{i_{av}(n+1)-i_{av}(n)}.$$

The duration of the pulse excitation signal is very short, and line voltage is regarded as a constant value, *V_g_*. In steady state, the output voltage, inductor current, and duty cycle are represented as *V*, *I_av_*, and *D*, respectively. Since the valley inductor current in the *n*th switching cycle is unchanged, $v_{L}(n)=0$, and (9) is provided by

(9) $$i_{av}(n)R_{L}=V_{g}D-V.$$

Therefore, (8) can be simplified as follows:

(10) $$\hat{L}=\frac{v_{x}(n+1)-v_{x}(n)}{i_{av}(n+1)-i_{av}(n)},$$

where $v_{x}(n)=[2d(n)-d^{2}(n)-D]V_{g}T/2$.

As shown in (10), a precise average inductor current is required for inductance monitoring. However, for a buck converter under SCM control, it is difficult to obtain inductor current accurately because the conventional current estimator is susceptible to inductance and parasitic parameter variations.

The accurate value of *i_av_*(*n* + 1) − *i_av_*(*n*) is derived by analyzing the capacitor charge balance principle.

According to the charging characteristics of the output capacitor, the relationship between capacitor voltage and inductor current can be described as follows:

(11) $$C\frac{v(n+2)-v(n+1)}{T}=i_{av}(n+1)-\frac{v(n+1)}{R}.$$

Then, the average inductor current of the *n*th switching cycle, $i_{av}(n)$, can obtained from (9).

(12) $$i_{av}(n)=C\frac{v(n+1)-v(n)}{T}+\frac{v(n)}{R}$$

Similarly, the average inductor current of the (*n* + 1)th switching cycle is provided by

(13) $$i_{av}(n+1)=C\frac{v(n+2)-v(n+1)}{T}+\frac{v(n+1)}{R}.$$

As Figure 2 shows, the system stays in a steady state in the *n*th cycle; thus, v(n+1)=v(n). Subtracting (12) from (13) yields

(14) $$i_{av}(n+1)-i_{av}(n)=\frac{C}{T}\left[v(n+2)-v(n+1)\right].$$

With (14), the variation in average inductor current required for inductance monitoring can be calculated using the sampled output voltage.

Combining (10) and (14), a current-sensorless inductance calculation model for the buck converter can be obtained, shown as follows:

(15) $$\hat{L}=\frac{v_{x}(n+1)-v_{x}(n)}{C[v(n+2)-v(n+1)]}T.$$

Equation (15) shows that only the duty cycle, line voltage, and output voltage are required for inductance online monitoring.

## 3. Compensation Strategy for Capacitor ESR Effect

**Figure 3:**
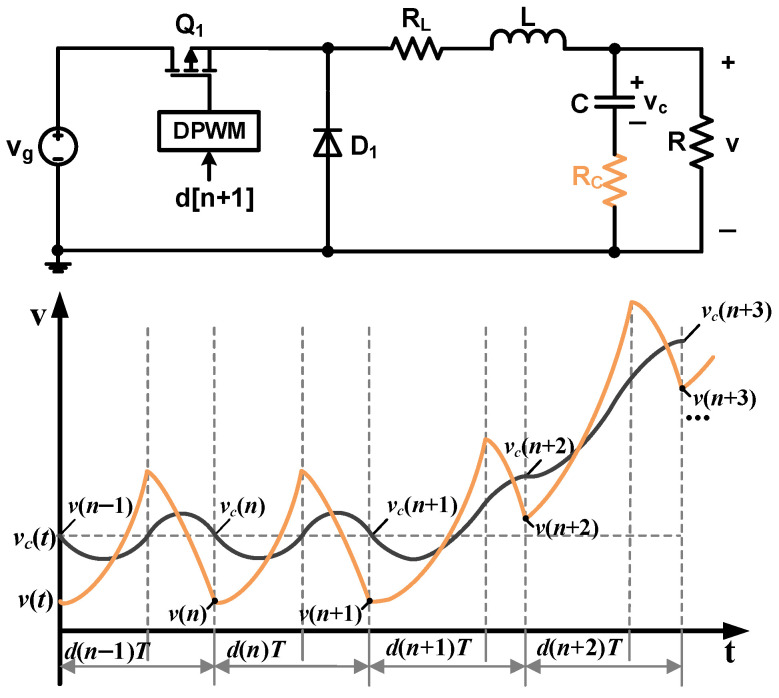
Buck converter model considering capacitor ESR and its capacitor and output voltages.

In the above derivation process, the output capacitor ESR is not considered. However, the capacitor ESR leads to differences between the sampled output voltage and capacitor voltage, as shown in Figure 3.

**Figure 4:**
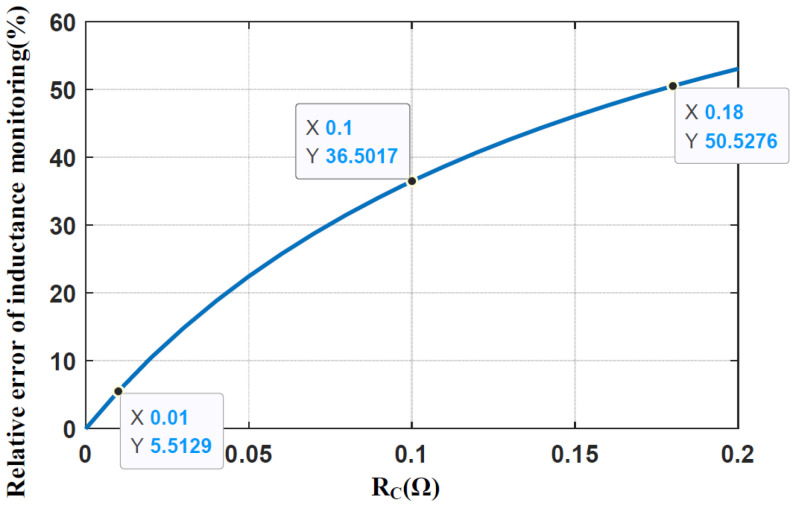
Simulation results for relative error in inductance monitoring under different *R_C_*.

The simulation results for different *R_C_* versus relative errors of inductance monitoring are shown in Figure 4, indicating that the monitoring error increases with *R_C_*. When *R_C_* < 0.01 Ω, the calculation error is less than 5.52%, which is acceptable. When *R_C_* = 0.1 Ω, the monitoring error rises to 36.5%, which means the effect of *R_C_* on inductance monitoring cannot be ignored. This error happens when ESR increases because of the aging effect or when a high ESR capacitor is used.

To improve monitoring accuracy, a compensation strategy for the effect of capacitor ESR on inductance calculation should be investigated. First, an inductance calculation model considering capacitor ESR is derived. Then, capacitor ESR is estimated to improve the accuracy of inductance monitoring.

### 3.1. Inductance Calculation Model Considering Capacitor ESR

As shown in Figure 3, the charging equation of the output capacitor is provided by

(16) $$C\frac{v_{C}(n+1)-v_{C}(n)}{T}=i_{av}(n)-\frac{v(n)}{R}.$$

The relationship between $v_{C}(n)$ and v(n) is

(17) $$v_{C}(n)=v(n)-\left[i_{v}(n)-\frac{v(n)}{R}\right]R_{C}.$$

Replacing $v_{C}$ in (16) with (17), the capacitor current during a switching cycle is described as follows:

(18) $$C\left(1+\frac{R_{C}}{R}\right)\frac{v(n+1)-v(n)}{T}-CR_{C}\frac{i_{v}(n+1)-i_{v}(n)}{T}=i_{av}(n)-\frac{v(n)}{R}.$$

As shown in (6), [*i_v_*(*n* + 1) − *i_v_*(*n*)]/*T* = *v_L_*(*n*)/*L*. Substituting it into (18), the average inductor current can be derived by

(19) $$i_{av}(n)=C\left(1+\frac{R_{C}}{R}\right)\frac{v(n+1)-v(n)}{T}-CR_{C}\frac{v_{L}(n)}{L}+\frac{v(n)}{R}.$$

Since v(n+1)=v(n) and $i_{v}(n+1)=i_{v}(n)$, the difference between $i_{av}(n)$ and $i_{av}(n+1)$ can be derived by

(20) $$i_{av}(n+1)-i_{av}(n)=C\left(1+\frac{R_{C}}{R}\right)\frac{v(n+2)-v(n+1)}{T}-CR_{C}\frac{v_{L}(n+1)}{L}.$$

Substituting (20) into (8), the inductance calculation model considering the *R_C_* effect is provided by

(21) $$\hat{L}=\frac{v_{x}(n+1)-v_{x}(n)+CR_{C}v_{L}(n+1)}{C\left(1+\frac{R_{C}}{R}\right)\left[v(n+2)-v(n+1)\right]}T,$$

where $v_{L}(n+1)=V_{g}[d(n+1)-D]$.

### 3.2. Capacitor ESR Estimation

To accurately compensate for the capacitor ESR effect and obtain a precise inductance monitoring result, an *R_C_* estimation method is investigated.

By substituting (8) into (20), the charging equation of the output capacitor in the (*n* + 1)th cycle can be rewritten as follows:

(22) $$C\left(1+\frac{R_{C}}{R}\right)\frac{v(n+2)-v(n+1)}{T}=CR_{C}\frac{v_{L}(n+1)}{L}+\frac{v_{x}(n+1)-v_{x}(n)}{L}.$$

Similarly, the current equation of the output capacitor in the (*n* + 2)th cycle approximates

(23) $$C\left(1+\frac{R_{C}}{R}\right)\frac{v(n+3)-v(n+2)}{T}=CR_{C}\frac{v_{L}(n+2)}{L}+\frac{v_{L}(n+1)T}{L}+\frac{v_{x}(n+2)-v_{x}(n)}{L}.$$

Let Δv(n+2)=v(n+2)−v(n+1). From (22) and (23), *R_C_* is provided by

(24) $${\hat{R}}_{C}=\frac{\Delta v(n+2)\left[v_{x}(n+2)-v_{x}(n)+v_{L}(n+1)T\right]-\Delta v(n+3)\left[v_{x}(n+1)-v_{x}(n)\right]}{C\left[\Delta v(n+3)v_{L}(n+1)-\Delta v(n+2)v_{L}(n+2)\right]}.$$

With the acquired data (line voltage, output voltage, and duty cycle) in the dynamic and steady states, the output capacitor ESR can be calculated, guaranteeing the accuracy of inductance monitoring.

## 4. Simulations

### 4.1. Robustness Analysis in Discrete-Time Domain

To validate the importance of inductance monitoring, a simulation system for the SCM-controlled buck converter was built in Matlab R2018b. Its main specifications are as follows: *v_g_* = 10 V, *v* = 6 V, *f* = 100 kHz, *L* = 57 μH, *C* = 22 μF, and *R_L_* = 0.12 Ω. For comparison, the influence of inductance variation on the system with and without inductance monitoring is analyzed.

**Figure 5:**
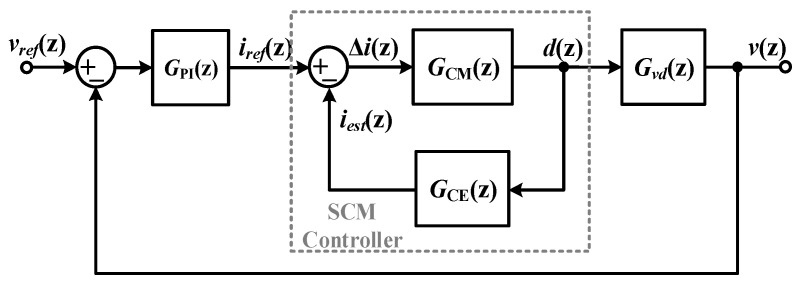
Closed-loop control diagram of SCM-controlled buck converter.

The closed-loop control system can be modeled as in Figure 5. The open-loop transform function and closed-loop transfer function of the SCM-controlled buck converter are expressed as (25) and (26), respectively.

(25) $$T(z)=G_{PI}(z)G_{di}(z)G_{vd}(z),$$

(26) $$\Phi (z)=\frac{G_{PI}(z)G_{di}(z)G_{vd}(z)}{1+G_{PI}(z)G_{di}(z)G_{vd}(z)},$$

where the transfer function of the PI controller is $G_{PI}(z)=K_{P}\left[1+T/T_{i}(z-1)\right]$.

By using Euler’s transformation method, the z-domain transfer function of the buck converter from *d* to *v* can be expressed as follows:

(27) $$G_{vd}(z)=\frac{v_{g}RT^{2}}{RLCz^{2}+(a-2RLC)z+(R+R_{L})T^{2}-a+RLC},$$

where $a=(R_{L}RC+L)T$.

Based on the modeling method in [25], the z-domain transfer function of the SCM controller can be derived, which is shown as follows:

(28) $$G_{di}(z)=\frac{\hat{d}}{{\hat{i}}_{ref}}=\frac{L^{\prime }(z^{2}-1)/T+2R_{L}}{z^{2}[(z+1)v_{g}-2G_{vd}(z)]},$$

where $L^{\prime }$ represents the inductance set in the SCM controller.

For a conventional SCM controller, $L^{\prime }$ is set to a fixed value, and the control performance may be degraded by inductance variation, whereas the proposed algorithm is used to update $L^{\prime }$ with the actual value. When inductance changes, the robustness difference between the systems with and without inductance monitoring can be investigated.

**Figure 6:**
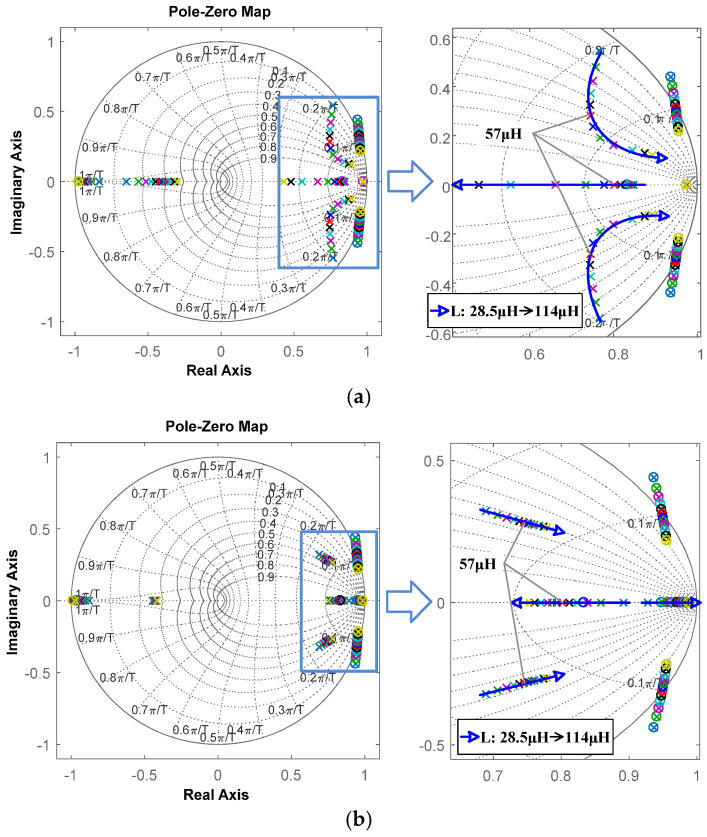
Migration of pole-zeros of SCM-controlled buck converter: (**a**) without inductance monitoring versus *L*; (**b**) with inductance monitoring versus *L*.

**Figure 7:**
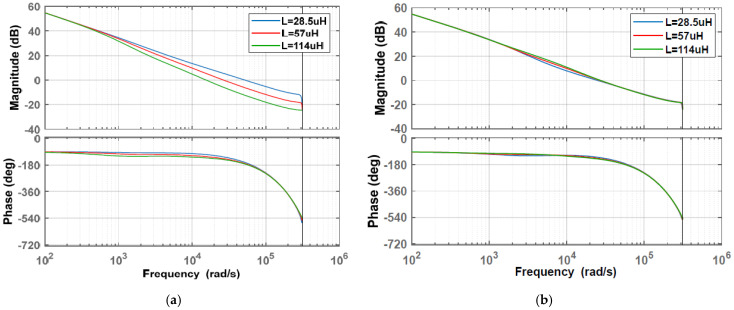
Open-loop Bode plot of SCM-controlled buck converter: (**a**) without inductance monitoring versus *L*; (**b**) with inductance monitoring versus *L*.

For the system without inductance monitoring, *L* in $G_{vd}(z)$ varies from 28.5 μH to 114 μH, while $L^{\prime }$ in the SCM controller is set as the nominal value (57 μH). The pole-zeros of the system and open-loop Bode plot under different *L* values are shown in Figure 6a and Figure 7a, respectively. As Figure 6a shows, the right figure is a partially enlarged detail of the left figure, and the blue arrow line represents the migration of the pole-zeros of the system when L in the buck circuit varies from 28.5 μH to 114 μH. As L deviates from the nominal value, poles are changed, and two of them move toward the boundary of the unit cycle, which means the transient response gradually degrades. The crossover frequency and phase margin from Figure 7a are listed in Table 1. When *L* changes to 28.5 µH, the crossing frequency nearly doubles increases, and the phase margin decreases to 17°. This means that the stability and transient response capability of the system without inductance monitoring have significantly decreased, which is consistent with the analysis in Figure 6a.

Comparatively, *L* and $L^{\prime }$ in the system with inductance monitoring are set to the same value, both changing from 28.5 μH to 114 μH. As shown in Figure 6b, the poles still change when *L* varies from 28.5 μH to 114 μH, but the pole change caused by *L* variation is much smaller, indicating that the system can maintain its high performance. The crossover frequency and phase margin of Figure 7b are also listed in Table 1. When *L* changes to 28.5 µH/114 µH, the change in crossing frequency is very small, and the phase margin only decreases by a maximum of 7 degrees. Therefore, inductance monitoring can effectively reduce the effect of inductance variation on system stability and transient response capability.

### 4.2. Simulation Results with Load/Line Voltage Variations

To verify the effectiveness of inductance monitoring under different conditions and the transient performance of the buck converter with the proposed method, simulations can be carried out under load and line voltage step changes. For comparison, the system without inductance monitoring is also employed for testing.

*L* in the buck circuit is set to 28.5 μH to stimulate inductance drift, and $L^{\prime }$ in the digital controller is set to 57 μH, which is the nominal value. Table 2 presents the simulation results for inductance monitoring when load/line voltage changes are made. The relative error of inductance estimation is between 0.28% and 1.92%, indicating load/input voltage variations have little impact on the inductance monitoring simulation results.

**Figure 8:**
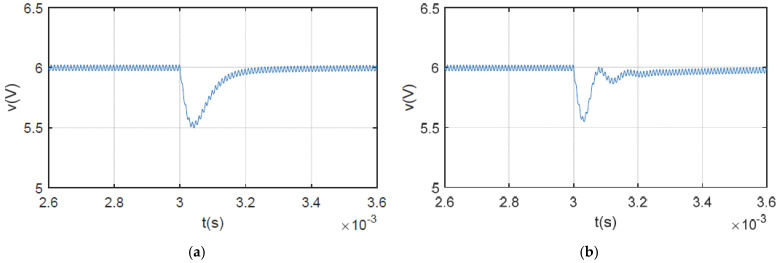
Simulation waveforms of output voltage when load step changes from 6 Ω to 4 Ω: (**a**) with proposed method; (**b**) without inductance monitoring.

**Figure 9:**
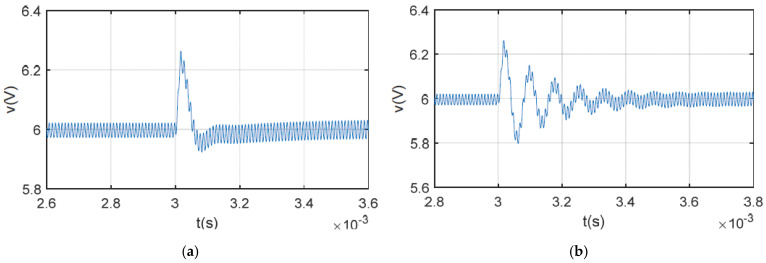
Simulation waveforms of output voltage when line voltage step changes from 10 V to 12 V: (**a**) with proposed method; (**b**) without inductance monitoring.

Figure 8 shows the simulation results for the output voltage when the load changes. As shown in Figure 8a, the SCM-controlled buck converter using the proposed method stabilizes within 220 μs. However, for the system without inductance monitoring, the output voltage recovers to 6 V with 330 μs, which is 50% longer. The simulation results for output voltage when the line voltage steps are presented in Figure 9. Ringing occurs in the system without inductance monitoring, causing the output voltage to take 600 μs to stabilize. The transient time is twice that of the system under the proposed algorithm. The simulation results shown in Figure 8 and Figure 9 are consistent with the robustness analysis in the previous section.

## 5. Experiments

**Figure 10:**
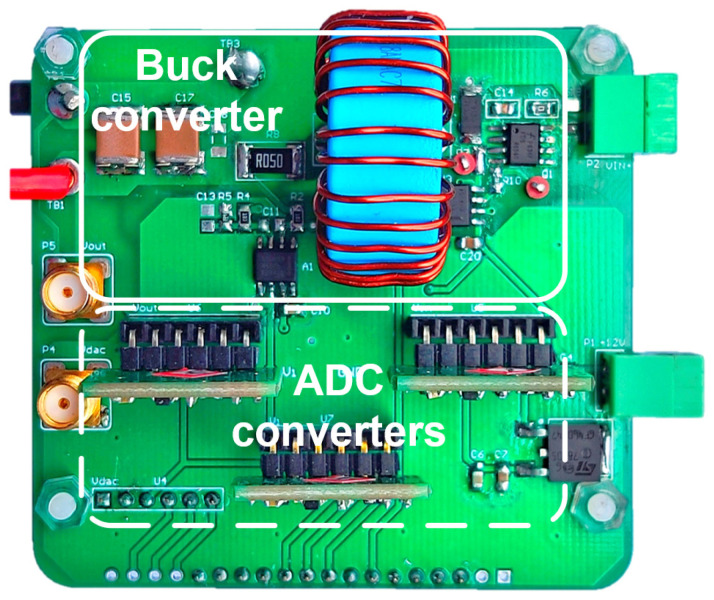
Photograph of experimental prototype.

A digitally controlled buck converter was constructed to validate the performance of the proposed method. The prototype is shown in Figure 10, and its specifications are consistent with that of the simulation. An FPGA (Cyclone IV) board was employed to implement the SCM controller and the proposed inductance monitoring method. For the main circuit, a MOSFET (FDS86540, ON Semiconductor, Phoenix, AZ, USA) and diode (NRVTSA4100E, ON Semiconductor, Phoenix, AZ, USA) were adopted as switching devices. CKG57NX7S2A226M500JH (TDK, Japan) was used as the output capacitor. The specifications are shown in Table 3. Two AD converter chips (LTC2314-14, Linear Technology, Milpitas, CA, USA) were used to sample line and output voltages.

Inductor parameters change with temperature, loading levels, aging effects, etc. Since the aging constant is very large, the inductance variations caused by aging effects are very small and last for a short time. To avoid wasting hardware resources, the period for inductance monitoring should not be set too short. While the temperature constant is much smaller than the aging constant and the load levels may change at any time, a short time period should be set to ensure timely monitoring. Considering these factors, inductance monitoring is performed once per second.

To verify the effectiveness of the proposed method, both buck converters with low and high capacitor ESR were employed for testing. Since the ESR of the output capacitor is small, a resistor (0.1 Ω) is connected in the series with the output capacitor to simulate high capacitor ESR.

### 5.1. Experimental Results with Low Capacitor ESR

#### 5.1.1. Inductance Monitoring Results

**Figure 11:**
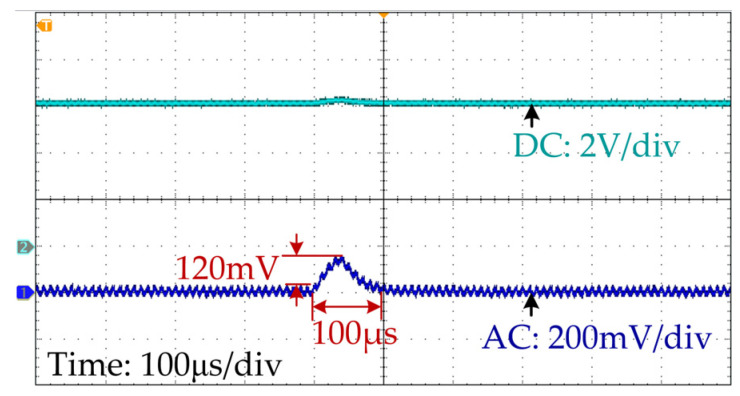
Output voltage waveform during pulse signal injection.

The output voltage waveform under pulse signal injection is shown in Figure 11. The amplitude and duration of Δ*v_ref_* are set as 3% of *V_ref_* and 40 μs, respectively. The duration of signal injection is chosen based on the transient time required for the inductance monitoring. The selection principles of the injection amplitude are as follows: (1) the amplitude should be large enough to reduce the influences of the output voltage measurement noises and quantization errors on the accuracy of inductance monitoring; (2) the disturbance caused by the signal injection will not greatly influence the system performance. The injection amplitude can be smaller if the system has a high-resolution AD converter.

As Figure 11 shows, the corresponding output voltage deviation (120 mV) is 2% of the nominal reference voltage, indicating that the impact of pulse injection on the system can be ignored. After the injection, the output voltage is restored to 6 V in 100 μs. During this period, the required line and output voltages are measured, and then, the inductance monitoring is processed.

**Figure 12:**
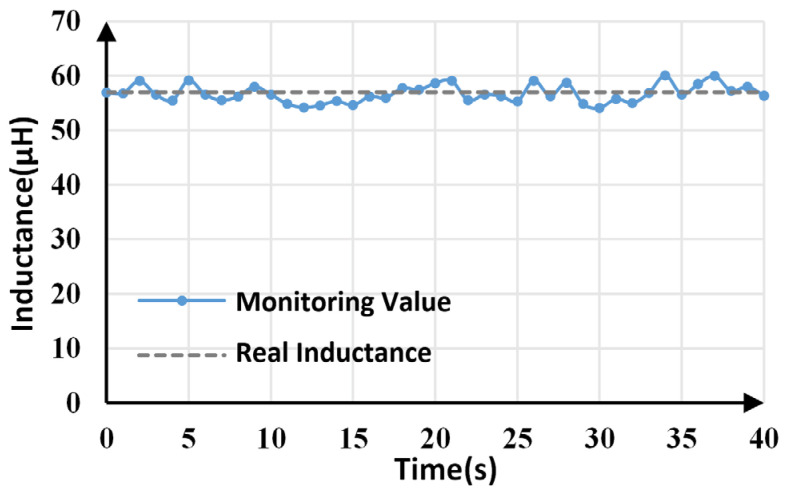
Inductance monitoring results.

Inductance monitoring results within 40 s are shown in Figure 12. The average monitoring value is 56.781 μH, which is very close to the actual value (57 μF). The maximum and minimum monitoring values are 60.098 μH and 54.115 μH, respectively, which means the maximum tracking error is less than 5.5%.

To verify the effectiveness of the proposed method when load and line voltage step changes occur, buck system experiments with/without inductance monitoring were also carried out.

#### 5.1.2. Experiment with Load Variations

**Figure 13:**
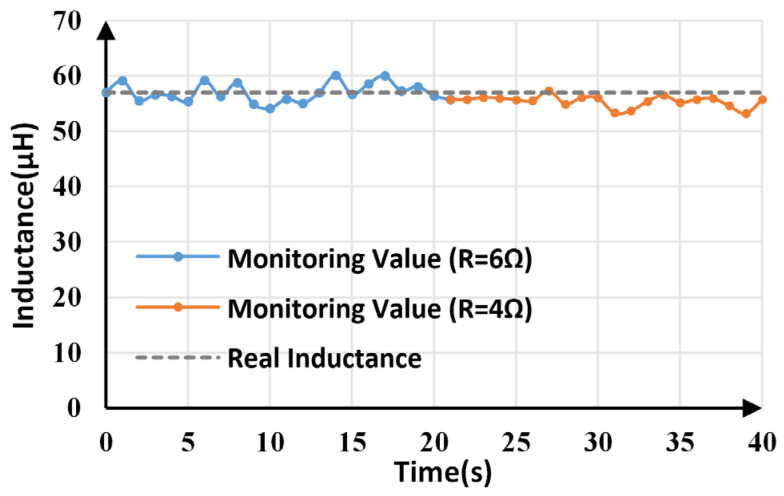
Inductance monitoring results under load variations.

When the load changes from 6 Ω and 4 Ω at 20.5 s, inductance monitoring results before and after the change are shown in Figure 13. For comparison, Table 4 lists the average, maximum, and minimum monitoring values of inductance under two different load conditions. The average value is still close to the actual value, with a maximum error of less than 6%, even though load resistance has changed.

**Figure 14:**
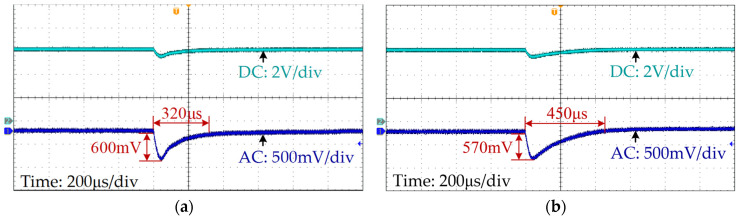
Output voltage waveforms when load steps change from 6 Ω to 4 Ω: (**a**) with proposed method; (**b**) without inductance monitoring.

With the proposed inductance monitoring, the output voltage response waveform of the SCM-controlled buck converter under load variations is shown in Figure 14a. When the load changes from 6 Ω to 4 Ω, the output voltage deviates by 600 mV and then reverts back to a steady state in 320 μs. However, for the SCM controller without inductance monitoring, the output voltage of the system stabilizes within 450 μs. The response time is 40.6% longer than that of the SCM controller with the proposed method.

#### 5.1.3. Experiment with Line Voltage Variations

**Figure 15:**
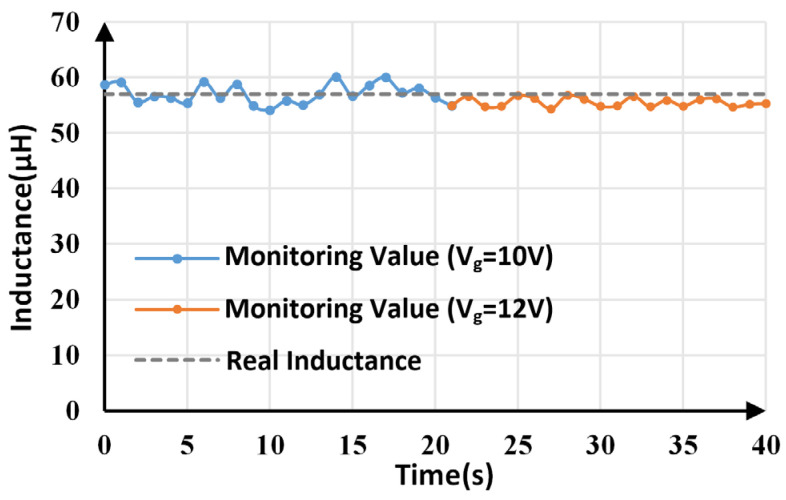
Inductance monitoring results under line voltage variations.

The line voltage was set to change from 10 V to 12 V at 20.5 s, and the corresponding inductance monitoring results are shown in Figure 15. The average, maximum, and minimum values of inductance calculated under different line voltages are provided in Table 5. Although the line voltage changes, the average value is slightly smaller than the actual value, and the maximum error is less than 5%.

**Figure 16:**
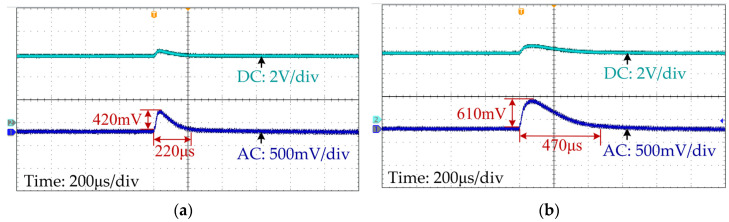
Output voltage waveforms when the line voltage step changes from 10 V to 12 V: (**a**) with proposed method; (**b**) without inductance monitoring.

The output voltage waveforms of the system with the proposed method and without inductance monitoring when line voltage rises from 10 V to 12 V are shown in Figure 16a and Figure 16b, respectively. As shown in Figure 16a, for the system with the proposed method, the output voltage deviates by 420 mV and takes 220 μs to stabilize, while for the SCM controller without inductance monitoring, the stabilization time of the output voltage is 470 μs, shown as Figure 16b, which is 113.6% longer.

The experimental results show that the proposed inductance monitoring method has reliable accuracy under different loads and line voltages. By updating the inductance in the controller, the system can achieve a faster response speed than systems without inductance monitoring when a sudden change occurs in the load or line voltage.

### 5.2. Experimental Results with High Capacitor ESR

To verify the effectiveness of the proposed method in high *R_C_* situations, experiments are executed with and without *R_C_* effect compensation. The experimental results show that the accuracy of inductance monitoring is effectively improved by the proposed compensation strategy.

**Figure 17:**
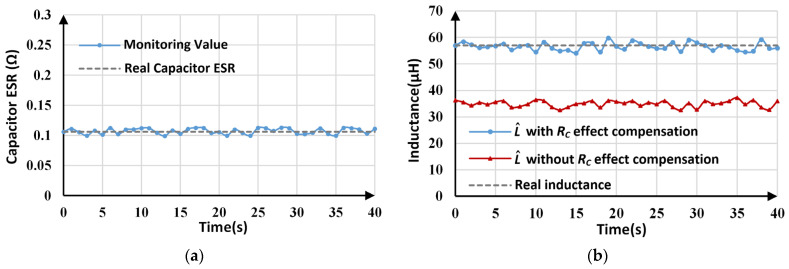
Estimated parameters with high capacitor ESR: (**a**) estimated *R_C_*; (**b**) estimated *L*.

The online monitoring results for *R_C_* and *L* are shown in Figure 17a and Figure 17b, respectively. As Figure 17a shows, the average value (0.107 Ω) of the estimated *R_C_* is very close to the actual value (0.106 Ω). The estimation results vary from 0.099 Ω to 0.113 Ω, which is caused by sampling and quantization errors. The effectiveness of *R_C_* estimation is reflected by the inductance monitoring results. As shown in Figure 17b, the inductance monitoring results without *R_C_* effect compensation fluctuate around 34.933 µH, which deviates from the actual value by 38.71%, while for inductance monitoring with *R_C_* effect compensation, the maximum tracking error is only about 5%, indicating that *R_C_* estimation and compensation effectively improve inductance monitoring accuracy.

**Figure 18:**
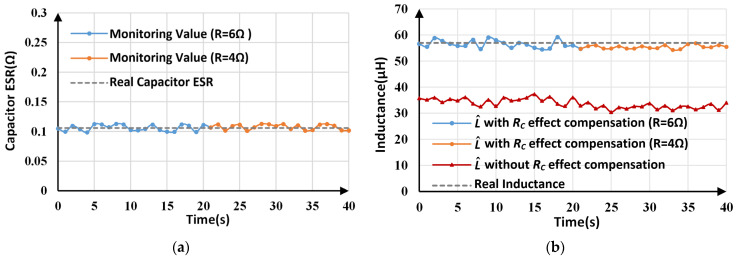
Estimated parameters for proposed method under load variations: (**a**) estimated *R_C_*; (**b**) estimated *L*.

**Figure 19:**
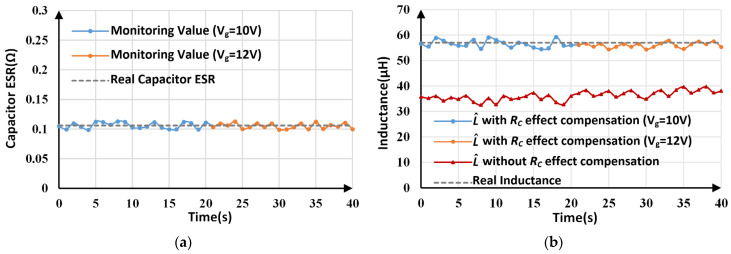
Estimated parameters by proposed method under line voltage variations: (**a**) estimated *R_C_*; (**b**) estimated *L*.

The monitoring results for *R_C_* and *L* under load and line voltage variations are depicted in Figure 18 and Figure 19, respectively. For $\hat{L}$ with *R_C_* effect compensation, a comparison of the experimental results before and after the load/line voltage changes is listed in Table 6. When the load changes to 4 Ω, the *R_C_* estimation results change between 0.101 Ω and 0.113 Ω, with an average value of 0.108 Ω. By using the estimated *R_C_* to inductance calculation, inductance monitoring results with an error of less than 5% are obtained. Similarly, when the line voltage changes to 12 V, the maximum inductance tracking error is about 4.57%.

These experimental results show that whether the system has low or high capacitor ESR, the proposed method can monitor the inductance with reliable accuracy.

### 5.3. Experimental Results with Different Switching Frequency

**Figure 20:**
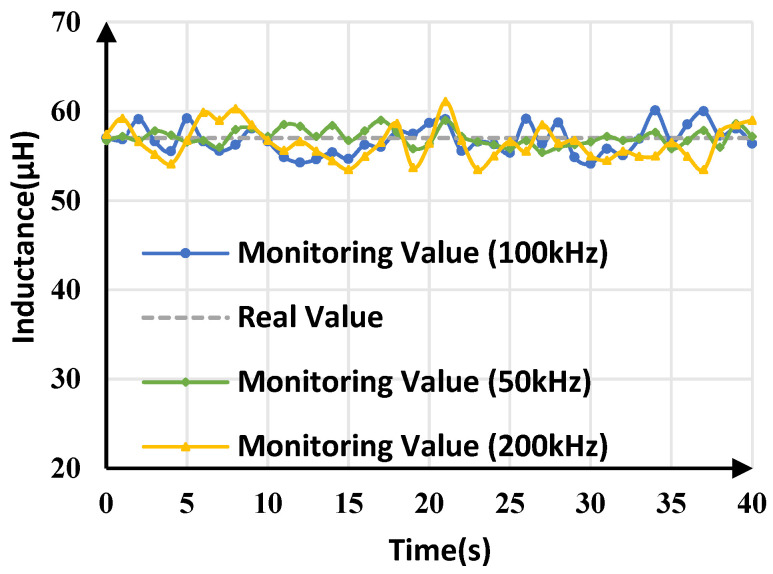
Inductance monitoring results for buck converters with different switching frequencies.

Buck converters with different switching frequencies have different transient states during signal injection, which may affect the accuracy of inductance monitoring. To investigate this effect, buck converters with different switching frequencies (50 kHz/200 kHz) were tested, and the inductance monitoring results are shown in Figure 20. As Table 7 shows, the average monitoring values of inductance at 50 kHz, 100 kHz, and 200 kHz are 57.089 μH, 56.781 μH, and 56.46 μH, respectively, and the monitoring errors are less than 1%. The maximum tracking error of inductance at 50 kHz is 3.53%, which is the smallest error among these three switching frequencies, while the maximum tracking error of inductance at 200 kHz is 7.20%, which is the highest. This indicates that as the switching frequency increases, the maximum monitoring error gradually increases. The reason is that at lower switching frequencies, the output voltage variations caused by signal injection increase, thereby reducing the impact of output voltage measurement noises and quantization errors on inductance monitoring.

### 5.4. Experimental Results with Different Inductors

**Figure 21:**
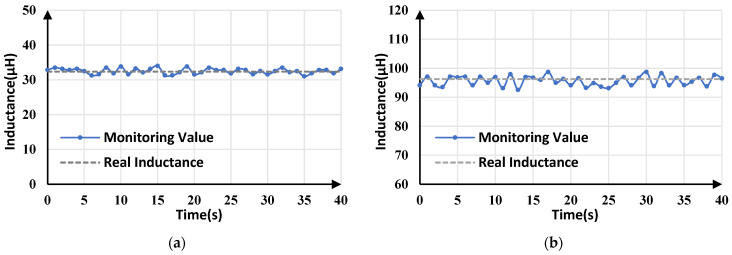
Inductance monitoring results for buck converters with different inductors: (**a**) *L* = 32.38 µH; (**b**) *L* = 96.28µH.

To verify the accuracy of the proposed algorithm under different inductance conditions, different inductors (33 µH/100 µH) were used for experimental testing. The inductance monitoring results are shown in Figure 21. The actual values of inductance were measured by an LCR meter, at 32.38 µH and 96.28 µH. The average monitoring values of Figure 21a,b are 32.52 µH and 95.628 µH, respectively, which are all close to the actual values. In Figure 21a, the maximum and minimum monitoring values are 33.839 μH and 3.021 μH, respectively. In Figure 21b, the maximum and minimum monitoring values are 98.702 μH and 92.541 μH, respectively. The maximum tracking error in both cases is less than 4.5%, meaning that the correlation between inductance estimation accuracy and inductance value is not significant.

## 6. Conclusions

This study presents an online inductance monitoring method for a buck converter without a current sensor. Based on dynamic characteristics with inductor volt-seconds and capacitor charge balance characteristics, a current-sensorless inductance calculation model is proposed. Considering aging and certain high ESR situations, the effect of capacitor ESR on inductance calculation is compensated to improve the monitoring accuracy. Only the line and output voltage need to be sampled, and the sampling frequency is much lower than in other inductance monitoring methods. With the monitored inductance, the parameters of the SCM controller for the buck converter can be updated. The simulation and experimental results show the effectiveness of the proposed method in inductance monitoring accuracy, improving the dynamic performance.

This research is ongoing, including monitoring methods for other topologies, multiparameter identification, monitoring accuracy and anti-interference capability improvement, and thermal testing, and we regret that the final results have not been fully obtained.

## Figures and Tables

**Table 1:** The crossover frequency and phase margin of the buck system.

*L*	Without Inductance Monitoring		With Inductance Monitoring	
	Crossover Frequency	Phase Margin	Crossover Frequency	Phase Margin
57 µH	4.25 kHz	45°	4.25 kHz	45°
28.5 µH	8.28 kHz	17°	3.92 kHz	52°
114 µH	2.48 kHz	48°	4.35 kHz	38°

**Table 2:** Comparison of monitoring simulation results when load/line voltage changes occur.

Load	Line Voltage	$\hat{L}$	Relative Error
6 Ω	10 V	28.30 µH	0.70%
4 Ω	10 V	27.95 µH	1.92%
6 Ω	12 V	28.42 µH	0.28%

**Table 3:** Specifications of tested buck converter.

*L*	*R*	*C*	*R_C_*	*R*	*V_D_*	*R_DS_*
57 μH	0.12 Ω	22 µF	0.006 Ω	6 Ω	0.45 V	0.011 Ω

**Table 4:** Comparison of inductance monitoring when load changes occur.

Load	$\hat{L}$ (avg)	Average Error	$\hat{L}$ (max)	$\hat{L}$ (min)	Maximum Error
6 Ω	56.781 µH	0.38%	60.098 µH	54.115 µH	5.44%
4 Ω	55.659 µH	2.35%	57.309 µH	53.676 µH	5.83%

**Table 5:** Comparison of inductance monitoring when line voltage changes occur.

Line Voltage	$\hat{L}$ (avg)	Average Error	$\hat{L}$ (max)	$\hat{L}$ (min)	Maximum Error
10 V	56.781 µH	0.38%	60.098 µH	54.115 µH	5.44%
12 V	55.505 µH	2.62%	56.751 µH	54.334 µH	4.68%

**Table 6:** Comparison of monitoring results when load/line voltage changes.

Load	Line Voltage	$\hat{L}$ (avg)	Average Error	$\hat{L}$ (max)	$\hat{L}$ (min)	Maximum Error	${\hat{R}}_{C}$ (avg)	Average Error
6 Ω	10 V	56.571 µH	0.75%	59.768 µH	54.129 µH	5.04%	0.107 Ω	0.94%
4 Ω	10 V	55.391 µH	2.82%	56.848 µH	54.306 µH	4.73%	0.108 Ω	1.89%
6 Ω	12 V	56.024 µH	1.71%	57.820 µH	54.386 µH	4.57%	0.104 Ω	1.89%

**Table 7:** Comparison of inductance monitoring at different switching frequencies.

Switching Frequency	$\hat{L}$ (avg)	Average Error	$\hat{L}$ (max)	$\hat{L}$ (min)	Maximum Error
50 kHz	57.089 μH	0.16%	59.012 µH	55.392 µH	3.53%
100 kHz	56.781 µH	0.38%	60.098 µH	54.115 µH	5.44%
200 kHz	56.460 μH	0.95%	61.106 µH	53.463 µH	7.20%

**Figure A1:**
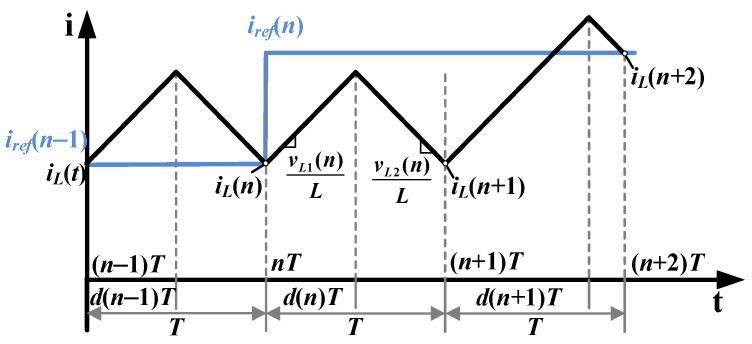
Inductor current waveforms of buck converter with CM controller.

**Figure A2:**
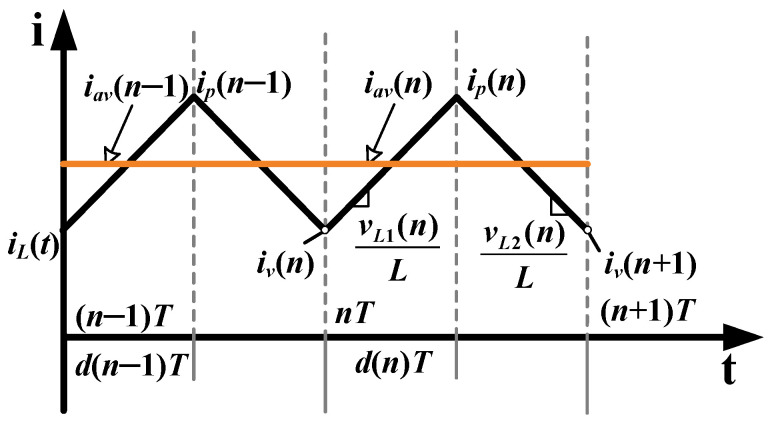
Inductor current waveforms.

## Data Availability

The data are contained within the article.

## Appendix A

### Appendix A.1

The current estimator and CM controller are derived as follows:

When the MOSFET switch Q_1_ is on, considering the parasitic parameter, R_L_, the inductor voltage, $v_{L1}(t)$, can be expressed as follows:

(A1) $$L\frac{di_{L}(t)}{dt}=v_{g}(t)-v(t)-i_{L}(t)R_{L}=v_{L1}(t).$$

During the switch-off period, the inductor voltage, $v_{L2}(t)$, can be rewritten as follows:

(A2) $$L\frac{di_{L}(t)}{dt}=-v(t)-i_{L}(t)R_{L}=v_{L2}(t).$$

Therefore, the variation in inductor current within one switching cycle can be calculated as follows:

(A3) $$L\frac{di_{L}(t)}{dt}=v_{L1}(t)d(t)+v_{L2}(t)d^{\prime }(t)=v_{g}(t)d(t)-v(t)-i_{L}(t)R_{L}.$$

By using the forward Euler method, (A3) can be discretized as follows:

(A4) $$i_{L}(n+1)=i_{L}(n)+\frac{T}{L}[v_{g}(n)d(n)-v(n)-i_{L}(n)R_{L}].$$

The control principle of the CM controller is described in Figure A1. When the reference inductance current, *i_ref_*, changes at nT time, the duty cycle of the (n + 1)th switching cycle can be computed in the nth switching cycle, making the inductor current follow the reference current at (n + 2)T time.

Similarly to (A4), the inductor current of the (n + 2)th switching cycle is expressed as follows:

(A5) $$i_{L}(n+2)=i_{L}(n+1)+\frac{T}{L}[v_{g}(n+1)d(n+1)-v(n+1)-i_{L}(n)R_{L}].$$

*d*(*n*) is obtained in the (n − 1)th switching cycle, which is still the steady state duty cycle. Therefore, v(n+1)=v(n). *v_g_* (n + 1) can be regarded as equal to *v_g_* (n + 1) because the line voltage changes slowly. Thus, (A5) can be rewritten as follows:

(A6) $$i_{L}(n+2)=i_{L}(n+1)+\frac{T}{L}[v_{g}(n)d(n+1)-v(n)-i_{L}(n)R_{L}].$$

Supposing $i_{L}(n+2)=i_{ref}(n)$, *d*(*n* + 1) can be calculated by

(A7) $$d(n+1)=\frac{L[i_{ref}(n)-i_{L}(n+1)]+[v(n)+i_{L}(n)R_{L}]T}{v_{g}(n)T}.$$

### Appendix A.2

According to the geometric characteristics of the inductor current, the relationship between $i_{v}(n)$ and $i_{av}(n)$ can be derived in detail (see Figure A2).

The rising and falling slopes of the inductor current within one switching cycle can be regarded as constants. The average inductor current, $i_{av}(n)$, can be computed by

(A8) $$i_{av}(n)=\frac{\left[i_{v}(n)+i_{p}(n)]d(n)+[i_{p}(n)+i_{v}(n+1)]d^{\prime }(n\right)}{2}$$

where $i_{v}(n)$ and $i_{p}(n)$ denote the valley and peak inductor currents of the nth switching cycle, respectively. $i_{p}(n)$ and $i_{v}(n+1)$ can be further expressed as (A9) and (A10).

(A9) $$i_{p}(n)=i_{v}(n)+\frac{v_{L1}(n)}{L}d(n)T$$

(A10) $$i_{v}(n+1)=i_{v}(n)+\frac{v_{L1}(n)}{L}d(n)T-\frac{v_{L2}(n)}{L}d^{\prime }(n)T$$

Substitute (A9) and (A10) into (A8), and (A8) can be rewritten as follows:

(A11) $$\begin{array}{cc} i_{v}(n) & =i_{av}(n)-\frac{T}{2L}\left[v_{L1}(n)d(n)d^{\prime }(n)+v_{L1}(n)d(n)-v_{L2}(n){d^{\prime }}^{2}(n)\right]\\ & =i_{av}(n)-\frac{v_{x}(n)}{L}, \end{array}$$

where $v_{x}(n)=[v_{L1}(n)d(n)d^{\prime }(n)+v_{L1}(n)d(n)-v_{L2}(n){d^{\prime }}^{2}(n)]T/2$.
